# Characterization of putative IgE-reactive N-terminal peptides from the 7S vicilin-like globulin of English Walnut (*Juglans regia*)

**DOI:** 10.1186/2045-7022-3-S3-P100

**Published:** 2013-07-25

**Authors:** M Downs, J Baumert, S Taylor, ENC Mills

**Affiliations:** 1 University of Manchester, Manchester, UK; 2 Food Allergy Research & Resource Program, University of Nebraska-Lincoln, Lincoln, NE, USA

## Background

The 7S vicilin-like seed storage globulins represent an important class of food allergens. In many plants, the 7S globulins are synthesized as a precursor polypeptide, which undergoes post-translational proteolysis to remove a portion of the N-terminus to produce the mature protein sequence. However, the fate of the cleaved N-terminal region is largely unknown. We have identified and characterized putative IgE-binding peptides derived from the 7S vicilin N-terminus in English walnut (*Juglans regia*).

## Methods

The low-molecular weight (LMW) protein fraction from raw, defatted English walnuts (cv. Chandler) was purified using Con-A sepharose, gel filtration, and anion exchange chromatography. One-dimensional polyacrylamide gel electrophoresis (1D-PAGE) was performed under non-reducing and reducing conditions. In-gel trypsin digestion followed by LC-MS/MS (LTQ Orbitrap™ XL) was used to identify protein bands of interest. The intact masses of proteins under native and reduced/alkylated conditions were evaluated with matrix-assisted laser desorption/ionization time-of-flight mass spectrometry (MALDI-TOF MS).

## Results

Two distinct 1D-PAGE patterns were observed in the purified LMW protein fraction. The first protein band pattern was indicative of a 2S albumin, but the second pattern represented an unknown protein. In-gel trypsin digestion and LC-MS/MS of the unknown bands revealed that they contained peptide sequences corresponding to the 7S vicilin-like protein from English walnut. The matched peptides, however, were located exclusively in the N-terminal region of the protein precursor sequence. Subsequent MALDI-TOF MS analysis of the protein fractions demonstrated a mass shift of 232 Da between the native and reduced/alkylated spectra, indicating the presence of four cysteine residues that form intramolecular disulfide bonds.

## Conclusion

Peptides cleaved from the N-terminal region of the 7S vicilin precursor are present in an intact form in walnut. IgE reactivity to similar peptides from walnut has been demonstrated in previous studies. Therefore, the mature 7S protein and these newly-described 7S N-terminal peptides represent two distinct populations of potential allergens, a fact which has substantial implications for protein sequences utilized for component-resolved diagnosis and allergen detection. Since the processing of 7S vicilin-like proteins has not been thoroughly elucidated in most plants, these types of protein fragments may be present in other nuts, seeds, and legumes.

## Disclosure of interest

None declared.

